# Complete genome sequence of *Methylomonas defluvii* YC3 isolated from the cyclic activated sludge system of a banknote printing facility

**DOI:** 10.1128/mra.01383-25

**Published:** 2026-05-18

**Authors:** Jiayan Li, Jie Zhao, Liyan Chen, Hang Yu

**Affiliations:** 1 College of Urban and Environmental Sciences, Peking University 105876, Beijing, China; 2 Unicorium Bio, Chengdu, Sichuan, China; University of Southern California, Los Angeles, California, USA

**Keywords:** methanotroph, genome, cyclic activated sludge system

## Abstract

We report the isolation and complete genome sequence of *Methylomonas defluvii* YC3 from the cyclic activated sludge system of a banknote-printing plant. The 5.2 Mbp genome comprises a single chromosome and three plasmids, and encodes key genes required for aerobic methane oxidation under both copper-replete and copper-limited conditions.

## ANNOUNCEMENT

*Methylomonas* spp. are type I methanotrophs in the family *Methylococcaceae* and are considered promising for methane-based bioconversion and the production of single-cell protein and value-added compounds from C1 substrates (1, 2). Here, we report the complete genome sequence of *Methylomonas defluvii* YC3, isolated from the cyclic activated sludge system (pH 8.12, 26.1°C, salinity < 0.2%; 30.6916°N, 103.8567°E) of a banknote-printing facility in Chengdu, Sichuan, China.

Enrichment was initiated by inoculating 1 mL of sludge into a 100 mL serum vial containing 20 mL of nitrate mineral salts (NMS) medium (DSMZ 921; pH 6.80) under a 1:4 methane:air atmosphere and incubating statically at 30°C for 15 days. The enrichment was transferred for three generations under the same conditions until pink turbidity and flocculation were observed, after which a pure isolate was obtained by streaking on NMS agar at 30°C. DNA was extracted from a liquid culture using a bacterial genomic DNA extraction kit (Majorbio, Shanghai, China). The 16S rRNA gene was amplified with primers Bac27F and U1492R and sequenced by the Sanger method (3). Genomic DNA was sequenced using both PacBio Sequel IIe and Illumina NovaSeq 6000 platforms. The same DNA extraction was used for both library preparations. For Illumina sequencing, DNA was sheared into 400–500 bp fragments using a Covaris M220 Focused Acoustic Shearer, and libraries were prepared using the NEXTflex Rapid DNA-Seq Kit (Bioo Scientific, USA) for 2 × 150 bp sequencing, generating 5,780,591 paired-end reads. For PacBio sequencing, DNA was fragmented into ~10 kb fragments using G-tubes (Covaris, MA, USA), purified, end-repaired, and ligated with SMRTbell adapters according to the manufacturer’s instructions, generating 59,809 HiFi reads (*N*_50_, 11,380 bp). Default parameters were used for all software unless otherwise specified. Illumina reads were quality filtered with fastp v0.23.4 (4), and clean reads were assembled with Unicycler v0.5.1 (5), and polished with Pilon v1.22 (6). Plasmids were annotated using PlasmidFinder v2.1.6 (7). The genome was circularized by trimming overlapping ends. Average nucleotide identity (ANI) was calculated using ANIcalculator v0.90 (8), and digital DNA-DNA hybridization (dDDH) was estimated using GGDC v3.0 (9). The genome was annotated using the NCBI Prokaryotic Genome Annotation Pipeline (PGAP v6.10) (10), and KEGG functional annotation was performed using KofamScan v1.3.0 (11).

**Fig 1:**
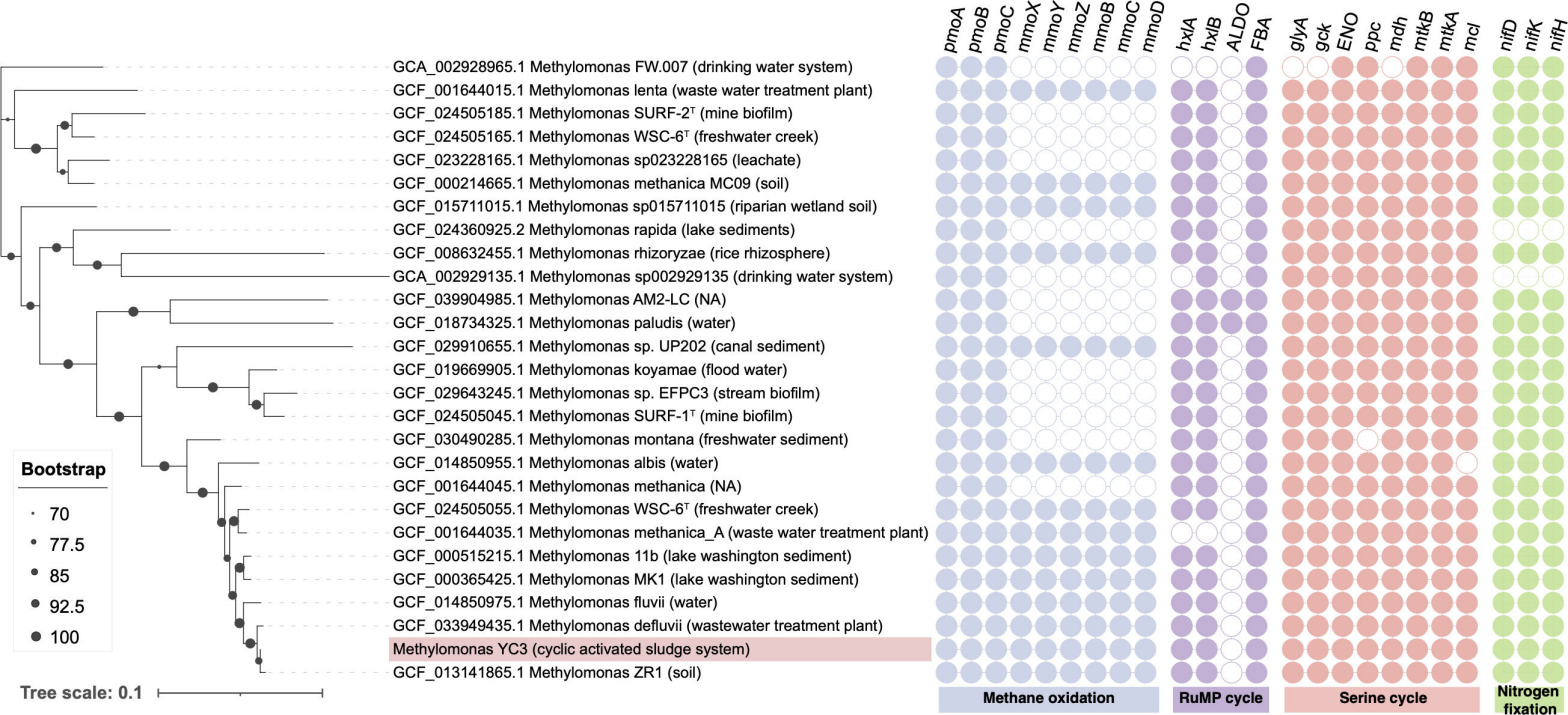
The maximum-likelihood tree constructed from a concatenated alignment of 120 bacterial marker genes (bac120) generated by GTDB-Tk v2.6.1 (13) from whole-genome sequences downloaded from GTDB, using IQ-TREE v2.4.0 with the Q.insect + F + I + R3 model selected by ModelFinder (14). Branch support was assessed with 1,000 ultrafast bootstrap replicates (values > 70% shown). The presence or absence of key genes (solid or open circles) was determined based on annotated KEGG IDs.

The YC3 genome comprises a single chromosome and three putative plasmids, with a total size of 5.2 Mbp and a G + C content of 51.5%, and encodes 8 rRNA operons, 47 tRNA genes, and 4,667 CDSs. YC3 is closest to *Methylomonas defluvii* OY6 (GCF_033949435.1) (12), with 99.43% 16S rRNA gene sequence identity, 95.60% ANI, and 63.70% dDDH value, supporting the assignment of YC3 to *Methylomonas defluvii*. YC3 is gram-negative and grows on methane or methanol as the sole carbon and energy source. Its genome encodes two particulate methane monooxygenase (pMMO) operons and one soluble methane monooxygenase (sMMO) operon, supporting aerobic methane oxidation under both copper-replete and copper-limited conditions (Fig. 1; Table 1).

**TABLE 1:** Genome characteristics and enzyme activity profiles of *Methylomonas defluvii* YC3^*a*^

Characteristics	YC3	Enzyme activity (API ZYM)	YC3	Enzyme activity (API 20NE)	YC3
Isolation source	The cyclic activated sludge system of a banknote printing facility	Water	−	NO_3_^−^	+
Total length (bp)	5,192,956	Alkaline phosphatase	+	NO_2_^−^	−
*N*_50_ (bp)	4,803,874	Esterase (C4)	+	Indole production	−
DNA G + C content, mol%	51.5	Esterase lipase (C8)	+	d-glucose fermentation	−
rRNA	8	Lipase (C14)	+	Arginine dihydrolase	−
tRNA	47	Leucine arylamidase	+	UREase	+
CDS	4,667	Valine arylamidase	+	β-glucosidase (esculin hydrolysis)	+
pMMO	+ (2)	Cystine arylamidase	+	Gelatin hydrolysis	−
sMMO	+	Trypsin	W	Beta-galactosidase	−
		α-chymotrypsin	−	d-glucose	−
		Acid phosphatase	W	l-arabinose	−
		Naphtol-AS-BI-phosphohydrolase	W	d-mannose	−
		α-galactosidase	−	d-mannitol	−
		β-galactosidase	−	N-acetyl-glucosamine	−
		β-glucuronidase	−	d-maltose	−
		α-glucosidase	−	Potassium gluconate	−
		β-glucosidase	+	Capric acid	−
		N-acetyl-β-glucosaminidase	−	Adipic acid	−
		α-mannosidase	−	Malic acid	−
		β-fucosidase	−	Trisodium citrate	−
				Phenylacetic acid	−

^*a*^ Analyses of enzyme activity profiles, gelatin and urease hydrolysis, and indole production were performed using API ZYM and API 20NE kits (bioMérieux). Enzyme activity: +, positive; W, weakly positive; −, negative.

## Data Availability

All raw sequence data have been deposited in the National Center for Biotechnology Information (NCBI) database under BioProject number PRJNA1363710 and BioSample number SAMN53233891, with corresponding Sequence Read Archive (SRA) accession number SRR36294272. The genome assembly has been deposited in GenBank under accession number GCA_054188105.1. The 16S rRNA gene sequence is available in GenBank under accession number PX485118. The KEGG annotation data has been deposited in Figshare: https://doi.org/10.6084/m9.figshare.32005896. All data sets are publicly accessible without restrictions.

## References

[1] Tikhonova EN, Suleimanov RZ, Miroshnikov KK, Oshkin IY, Belova SE, Danilova OV, Ashikhmin AA, Konopkin AA, But SY, Khmelenina VN, Pimenov NV, Dedysh SN. 2023. Methylomonas rapida sp. nov., a novel species of fast-growing, carotenoid-producing obligate methanotrophs with high biotechnological potential. Syst Appl Microbiol 46:126398. doi:10.1016/j.syapm.2023.126398 36724672

[2] Guo W, Li D, He R, Wu M, Chen W, Gao F, Zhang Z, Yao Y, Yu L, Chen S. 2017. Synthesizing value‐added products from methane by a new Methylomonas. J Appl Microbiol 123:1214–1227. doi:10.1111/jam.13581 28888065

[3] Yu H, Leadbetter JR. 2020. Bacterial chemolithoautotrophy via manganese oxidation. Nature 583:453–458. doi:10.1038/s41586-020-2468-5 32669693 PMC7802741

[4] Chen S. 2023. Ultrafast one-pass FASTQ data preprocessing, quality control, and deduplication using fastp. Imeta 2:e107. doi:10.1002/imt2.107 38868435 PMC10989850

[5] Wick RR, Judd LM, Gorrie CL, Holt KE. 2017. Unicycler: resolving bacterial genome assemblies from short and long sequencing reads. PLoS Comput Biol 13:e1005595. doi:10.1371/journal.pcbi.1005595 28594827 PMC5481147

[6] Walker BJ, Abeel T, Shea T, Priest M, Abouelliel A, Sakthikumar S, Cuomo CA, Zeng Q, Wortman J, Young SK, Earl AM. 2014. Pilon: an integrated tool for comprehensive microbial variant detection and genome assembly improvement. PLoS One 9:e112963. doi:10.1371/journal.pone.0112963 25409509 PMC4237348

[7] Carattoli A, Zankari E, García-Fernández A, Voldby Larsen M, Lund O, Villa L, Møller Aarestrup F, Hasman H. 2014. In silico detection and typing of plasmids using PlasmidFinder and plasmid multilocus sequence typing. Antimicrob Agents Chemother 58:3895–3903. doi:10.1128/AAC.02412-14 24777092 PMC4068535

[8] Yoon SH, Ha SM, Lim J, Kwon S, Chun J. 2017. A large-scale evaluation of algorithms to calculate average nucleotide identity. Antonie Van Leeuwenhoek 110:1281–1286. doi:10.1007/s10482-017-0844-4 28204908

[9] Meier-Kolthoff JP, Carbasse JS, Peinado-Olarte RL, Göker M. 2022. TYGS and LPSN: a database tandem for fast and reliable genome-based classification and nomenclature of prokaryotes. Nucleic Acids Res 50:D801–D807. doi:10.1093/nar/gkab902 34634793 PMC8728197

[10] Tatusova T, DiCuccio M, Badretdin A, Chetvernin V, Nawrocki EP, Zaslavsky L, Lomsadze A, Pruitt KD, Borodovsky M, Ostell J. 2016. NCBI prokaryotic genome annotation pipeline. Nucleic Acids Res 44:6614–6624. doi:10.1093/nar/gkw569 27342282 PMC5001611

[11] Aramaki T, Blanc-Mathieu R, Endo H, Ohkubo K, Kanehisa M, Goto S, Ogata H. 2020. KofamKOALA: KEGG Ortholog assignment based on profile HMM and adaptive score threshold. Bioinformatics 36:2251–2252. doi:10.1093/bioinformatics/btz859 31742321 PMC7141845

[12] Ouyang M-Y, Wang S, Nie W-H, Wang P-H, Liao W-X, Liu X-H, Lin S-S, Lin R-P, Chen G-Y, Zhu B, Shen J. 2024. Methylomonas defluvii sp. nov., a type I methane-oxidizing bacterium from a secondary sedimentation tank of a wastewater treatment plant. Int J Syst Evol Microbiol 74:006321. doi:10.1099/ijsem.0.006321 38607367

[13] Chaumeil P-A, Mussig AJ, Hugenholtz P, Parks DH. 2022. GTDB-Tk v2: memory friendly classification with the genome taxonomy database. Bioinformatics 38:5315–5316. doi:10.1093/bioinformatics/btac672 36218463 PMC9710552

[14] Minh BQ, Schmidt HA, Chernomor O, Schrempf D, Woodhams MD, von Haeseler A, Lanfear R. 2020. IQ-TREE 2: new models and efficient methods for phylogenetic inference in the genomic era. Mol Biol Evol 37:1530–1534. doi:10.1093/molbev/msaa015 32011700 PMC7182206

